# An Agile Systems Modeling Framework for Bed Resource Planning During COVID-19 Pandemic in Singapore

**DOI:** 10.3389/fpubh.2022.714092

**Published:** 2022-05-18

**Authors:** Sean Shao Wei Lam, Ahmad Reza Pourghaderi, Hairil Rizal Abdullah, Francis Ngoc Hoang Long Nguyen, Fahad Javaid Siddiqui, John Pastor Ansah, Jenny G. Low, David Bruce Matchar, Marcus Eng Hock Ong

**Affiliations:** ^1^Health Services and Systems Research, Duke-NUS Medical School, Singapore, Singapore; ^2^Health Services Research Centre, Singapore Health Services, Singapore, Singapore; ^3^SingHealth Duke NUS Academic Medical Centre, Health Services Research Institute, Singapore, Singapore; ^4^Lee Kong Chian School of Business, School of Computing and Information Systems, Singapore Management University, Singapore, Singapore; ^5^Department of Anesthesiology, Singapore General Hospital, Singapore, Singapore; ^6^Residential College 4, National University of Singapore, Singapore, Singapore; ^7^Department of Infectious Diseases, Singapore General Hospital, Singapore, Singapore; ^8^Programme in Emerging Infectious Diseases, Duke-NUS Medical School, Singapore, Singapore; ^9^Department of Internal Medicine (General Internal Medicine), Duke University Medical School, Durham, NC, United States; ^10^Department of Internal Medicine, Singapore General Hospital, Singapore, Singapore; ^11^Department of Emergency Medicine, Singapore General Hospital, Singapore, Singapore

**Keywords:** hospital bed management, systems dynamics modeling, COVID-19 pandemic, agile resource planning, agile resource allocation

## Abstract

**Background:**

The COVID-19 pandemic has had a major impact on health systems globally. The sufficiency of hospitals' bed resource is a cornerstone for access to care which can significantly impact the public health outcomes.

**Objective:**

We describe the development of a dynamic simulation framework to support agile resource planning during the COVID-19 pandemic in Singapore.

**Materials and Methods:**

The study data were derived from the Singapore General Hospital and public domain sources over the period from 1 January 2020 till 31 May 2020 covering the period when the initial outbreak and surge of COVID-19 cases in Singapore happened. The simulation models and its variants take into consideration the dynamic evolution of the pandemic and the rapidly evolving policies and processes in Singapore.

**Results:**

The models were calibrated against historical data for the Singapore COVID-19 situation. Several variants of the resource planning model were rapidly developed to adapt to the fast-changing COVID-19 situation in Singapore.

**Conclusion:**

The agility in adaptable models and robust collaborative management structure enabled the quick deployment of human and capital resources to sustain the high level of health services delivery during the COVID-19 surge.

## Introduction

With more than 102.1 million cases and 2.2 million deaths worldwide as of 31 January 2021 (1), the COVID-19 pandemic caused by the severe acute respiratory syndrome Coronavirus-2 virus (SARS CoV-2) is leading to substantial healthcare, economic, social and psychological impact. Even though the first cases of COVID-19 were confirmed in December 2019, the scientific world has just begun to understand better the health problems caused by this virus (2). Apart from the obvious medical issues, the pandemic has had both direct and indirect impact on healthcare demand, processes and outcomes. The many unknowns in such emerging infectious diseases would mean that governments and health systems around the world must react rapidly in the face of new information.

Sufficiency of healthcare capacity is a cornerstone for access to care which can significantly impact the public health outcomes for COVID-19. Many healthcare facilities around the world are seeing a surge in demand for hospital and intensive care unit (ICU) beds due to the pandemic. The case fatality rates (CFR) of COVID patients has shown to be significantly worse in overstretched health systems (3). Apart from containment and mitigation strategies that are important for “flattening” the infection curve, there is a critical need to ensure a resilient health system that can withstand unpredictable shocks resulting from the pandemic. Well-coordinated public and private sector policies and initiatives are essential to maintain high-quality care outcomes for the population (4). Globally, several healthcare systems have reported commendable efforts in planning and implementing surge capacities to cope with the on-going COVID-19 pandemic (5–8).

Most traditional epidemiological models developed for COVID-19 have focused on the demand side (e.g., flattening of the epidemic curve) (9–11). Such models may not fully capture nuanced disease outbreak scenarios together with policies and processes that are important to consider during pandemics (12). The projections from traditional epidemiological models are also fraught with uncertainties during the initial phases of a pandemic. This has shown to be the case when potential hotspots were difficult to be identified a priori to guide resource planning efforts (13). In Singapore, most of the early demand projections were based on importation and secondary local transmission models and were not able to predict the large clusters of positive cases that were discovered in migrant workers' dormitories (14). On the other hand, systems modeling techniques (e.g., system dynamics, SD, and discrete events simulation, DES) (15, 16) have seen many applications for both predictable demand patterns (17–19) and less predictable demand surges (e.g., disaster planning and pandemics due to emerging infectious diseases) (20–25). These simulation methods have the ability to capture both detailed system interactions (dynamic complexity) and structure (causal relationships) to provide a risk-free virtualized experimentation platform (26–29).

We describe the development of a dynamic simulation modeling framework to support agile bed capacity planning during the COVID-19 pandemic. A robust and effective data capture infrastructure within health systems, governance policies and modeling expertise embedded within the health systems are among the critical ingredients for success. To our knowledge, this is the first model that captured the nuanced COVID-19 response of the Singapore healthcare system. The model was further expanded to incorporate the national response, which included rapid flexing of bed capacity beyond the study hospital for informing other bed capacity planning needs for healthcare authorities.

## Materials and Methods

### Study Site and Data

Singapore is a city-state with a population size of ~5.7 million (of which 4 million are citizens and permanent residents) (28) with a land area of ~724 square km (29). Singapore had ~300,000 migrant workers working in the construction sector at the time of the study (30). In addition, Singapore had about 12,000 acute care beds in public and private hospitals (31) and ~400 ICU beds. In 2018, Singapore had a trained doctor-to-patient ratio of 2.4 doctors per 1,000 patients (32). The study hospital (SH) is Singapore General Hospital (SGH) which is the largest comprehensive public hospital in Singapore. SGH comprises of more than 30 clinical disciplines and ~1,700 inpatient beds. The hospital saw more than 25,000 surgeries and had 18 ICU beds in 2019. Singapore General Hospital is part of the Duke-NUS Medical School and Singapore Health Services (SingHealth) Academic Medicine Centre (AMC). A core resource of the AMC is an embedded data science unit in the Health Services Research Centre established since 2015.

The study data were derived from the Singapore General Hospital and public domain sources over the period from 1 January 2020 till 31 May 2020 covering the period when the initial outbreak and surge of COVID-19 cases in Singapore happened. Data used for estimating the model parameters consisted of 3 main sources: (1) daily hospital reports from the SH on the cumulative numbers of hospitalized, discharged, transferred to external facilities, death and the daily census of admissions; (2) situation reports and data consolidated by the AMC's disease outbreak task forces and the SH command center, and; (3) public domain data released by the Singapore Ministry of Health. Data with missing records and incomplete statistics were excluded from the analysis. While the first case of migrant worker infection was reported in January 2020, the scale of the COVID-19 outbreak was apparent around the end of March. Tracking of the flows from the migrant workers' dormitories started from 6th April 2020. Records containing data entry errors or duplicates were removed. All data were derived from government agency and public healthcare institutions for reporting purposes. There were multiple layers of data cleaning and veracity checks to ensure that data were sufficiently clean and robust for development of the models.

### Operational Policies in Study Site

Given the quick identification of the hotspots, the dormitory clusters in Singapore were contained and separated from the larger community and widespread community transmission was avoided. There have also been several dynamic adjustments made for the detection, diagnosis and disposition policies of patients. These include (polymerase chain reaction) PCR testing operations in the dormitories and community, heightened surveillance and targeted testing for vulnerable groups (33) and agile flexing of in-hospital and external isolation facilities (34). Some of the operational strategies in the SH and purpose-built facilities are summarized as follows.

(1) *Fever Screening Areas (FSA)* dedicated to the screening, triaging and disposition of COVID related cases. These facilities were planned and went operational on 20th March 2020 in the SH. FSA provides screening services for those who meet suspect case definition but do not yet require resuscitation care facilities. The dedicated FSA reduced the risk of cross-contamination of uninfected patients who required emergency care at the ED. FSA also played a key role in the implementation of the Swab and Send Home (SASH) Programme.
(2) *Swab and Send Home (SASH)*. The hospital's first point of contact (ED and FSA) take in patients with Covid-19 symptoms. At these nodes, the SASH policy allows some of the suspect cases who are at lower risk to be sent home for self-isolation to await result confirmation after the respiratory swab for SARS CoV-2 PCR test has been performed.
(3) *Types of beds for suspected and confirmed COVID-19 patients*. The types of beds that are designated for the care of suspected high-risk patients or confirmed COVID-19 patients are situated in the Acute Respiratory Infection (ARI) wards, Isolation (ISO) Wards and ICUs. Some of the beds for these wards were reconfigured from wards with 6 or 8 beds to house fewer beds per room (3 to 4 beds) to ensure there is sufficient distancing between beds. High risk suspect patients were placed in ARI wards. Patients who subsequently tested negative could be moved out of these ARI beds. ISO beds are isolation care facilities for confirmed cases. Patients from both ISO and ARI beds can be decanted to external community facilities to ensure hospital facilities are not overstretched.
(4) *Other surge capacities*. When the national Disease Outbreak Response System Condition (DORSCON) level was raised to “Orange,” the second highest alert status, from 7 February 2020 (35), the flexing of bed capacity and corresponding reduction of elective surgical loads were activated. ARI beds that were deployed to admit suspect and pneumonia patients were consolidated and ring-fenced. With the rapid rise in cases from the community and migrant dormitory outbreaks, the ISO capacity was also expanded. Partnerships with private hospitals and external large-scale facility operators, such as exhibition centers, military camps and port facilities rapidly expanded community isolation facilities (CIF) outside the hospitals from 500 to more than 40,000 beds progressively in phases.

### Modeling Framework

**Figure 1:**
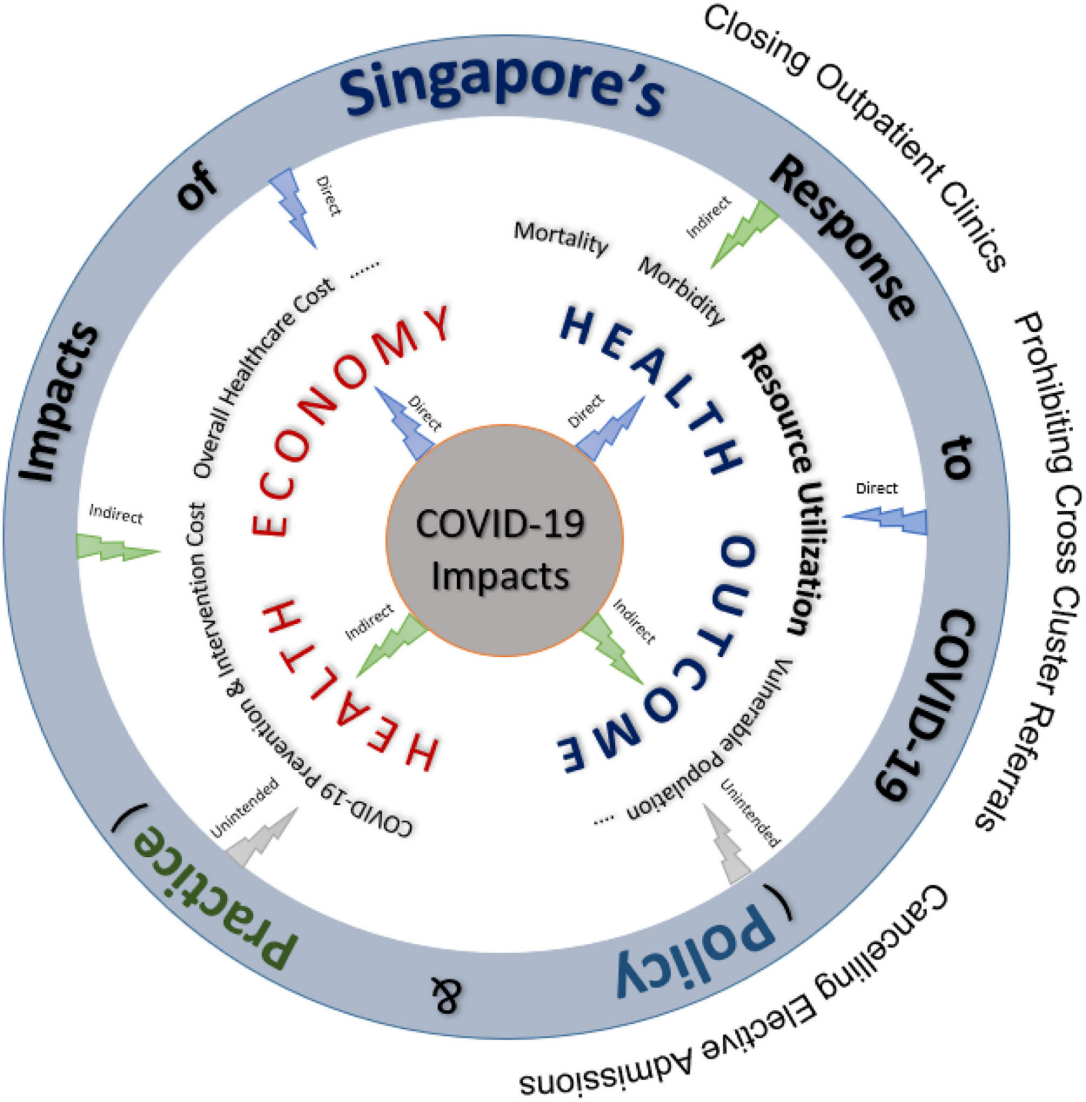
High-level Schematic of the Systems Modeling Framework in Singapore.

The bed resource modeling framework developed in this study provides estimates of mortality, morbidity, waiting times for specialist appointments and elective surgeries, length of stay (LOS), bed demand and utilization of critical hospital facilities (e.g., operating theaters, hospital and intensive care beds, isolation wards, diagnostic equipment, laboratory services). Figure 1 describes how the modeling framework is integrated within a larger ecosystem that looks at both the health economics and outcomes jointly to derive real-world insights into the impact of COVID-19 response strategies on the health system. The simulation models can be quickly calibrated to changes from both the demand and supply side for bed resource planning.

**Figure 2:**
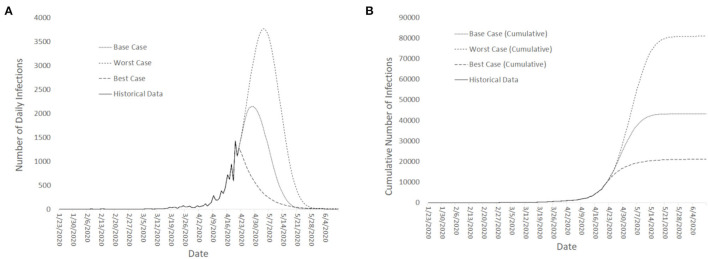
Short-term (month ahead) projections for: **(A)** Number of confirmed COVID-19 new cases, and; **(B)** Cumulative number of total COVID-19 Cases (in Singapore).

The arrival rates are estimated from the projected number of new cases at the national level, adjusted by the proportion of national demand seen by the study hospital (36). The demand module feeds the system with COVID-19 suspect community cases to the ED and FSA. Confirmed cases of age group *k* from dormitories were sent directly to ISO with a rate of $\gamma_{ISO}^{k}$. With reference to Figure 2, the following set of differential equations describes the journey of COVID-19 suspect/confirmed cases in Variants 2 and 3.


(1) $$\begin{array}{l} \frac{dED(t)}{dt}=\lambda_{ED}-(\lambda_{ED-SASH}+\lambda_{ED-FSA}+\lambda_{ED-ARI}+\lambda_{ED-ISO}\\ \;+\lambda_{ED-ICU})ED(t) \end{array}$$

(2) $$\begin{array}{l} \frac{dFSA(t)}{dt}=\lambda_{FSA}-(\lambda_{FSA-SASH}+\lambda_{FSA-ED}\\ \;+\lambda_{FSA-ARI})FSA(t) \end{array}$$

(3) $$\begin{array}{l} \frac{dSASH(t)}{dt}=\lambda_{ED-SASH}ED\left(t\right)+\lambda_{FSA-SASH}FSA\left(t\right)\\ \;-(\lambda_{SASH-ISO}+\lambda_{SASH-Neg})SASH(t) \end{array}$$

(4) $$\begin{array}{l} \frac{dARI(t)}{dt}=\lambda_{ED-ARI}ED\left(t\right)+\lambda_{FSA-ARI}FSA\left(t\right)\\ \;-(\lambda_{ARI-Neg}+\lambda_{ARI-ISO})ARI(t) \end{array}$$

(5a) $$\begin{array}{l} \frac{dISO_{c}\left(t\right)}{dt}=\lambda_{SASH-ISO}SASH\left(t\right)+\lambda_{ED-ISO}ED\left(t\right)\\ \;+\lambda_{FSA-ISO}FSA\left(t\right)+\lambda_{ICU-ISO}ICU_{c}\left(t\right)\\ \;-(\lambda_{ISO-Rec}+\lambda_{ISO-CIF}+\lambda_{ISO-ICU})ISO_{c}(t) \end{array}$$

(5b) $$\begin{array}{l} \frac{dISO_{d}^{k}\left(t\right)}{dt}=\gamma_{ISO}^{k}+\gamma_{CIF-ISO}^{k}CIF_{d}^{k}(t)+\gamma_{ICU-ISO}^{k}ICU_{d}^{k}(t)\\ \;-(\gamma_{ISO-Rec}^{k}+\gamma_{ISO-CIF}^{k}+\gamma_{ISO-ICU}^{k})ISO_{d}^{k}\left(t\right)\forall k\in G \end{array}$$

(6a) $$\begin{array}{l} \frac{dCIF_{c}(t)}{dt}=\lambda_{ISO-CIF}ISO\left(t\right)-(\lambda_{CIF-Rec}+\lambda_{CIF-ISO}\\ \;+\lambda_{CIF-ICU}+\lambda_{CIF-Mor})CIF_{c}(t) \end{array}$$

(6b) $$\begin{array}{l} \frac{dCIF_{d}^{k}(t)}{dt}=\gamma_{ISO-CIF}^{k}ISO_{d}^{k}\left(t\right)-(\gamma_{CIF-Rec}^{k}+\gamma_{CIF-ISO}^{k}+\gamma_{CIF-ICU}^{k}\\ \;+\gamma_{CIF-Mor}^{k})CIF_{d}^{k}(t)\;\forall k\in G \end{array}$$

(7a) $$\begin{array}{l} \frac{dICU_{c}(t)}{dt}=\lambda_{ED-ICU}ED\left(t\right)+\lambda_{ISO-ICU}ISO_{c}\left(t\right)\\ \;+\lambda_{CIF-ICU}CIF_{c}\left(t\right)-(\lambda_{ICU-ISO}\\ \;+\lambda_{ICU-Mor})ICU_{c}(t) \end{array}$$

(7b) $$\begin{array}{l} \frac{dICU_{d}^{k}(t)}{dt}=\gamma_{ISO-ICU}^{k}ISO_{d}^{k}\left(t\right)+\gamma_{CIF-ICU}^{k}CIF_{d}^{k}\left(t\right)\\ \;-(\gamma_{ICU-ISO}^{k}+\gamma_{ICU-Mor}^{k})ICU_{d}^{k}(t)\;\forall k\in G \end{array}$$

(8) $$\begin{array}{l} \frac{dR(t)}{dt}=\lambda_{ISO-Rec}ISO\left(t\right)+\lambda_{CIF-Rec}CIF\left(t\right)\\ \;+\sum_{k\in G}\left(\gamma_{ISO-Rec}^{k}ISO_{d}^{k}\left(t\right)+\gamma_{CIF-Rec}^{k}CIF_{d}^{k}\left(t\right)\right) \end{array}$$

(9) $$\begin{array}{l} \frac{dD(t)}{dt}=\lambda_{ISO-Mor}ISO\left(t\right)+\lambda_{CIF-Mor}CIF\left(t\right)\\ \;+\lambda_{ICU-Mor}ICU\left(t\right)+\sum_{k\in G}(\gamma_{ISO-Mor}^{k}ISO_{d}^{k}\left(t\right)\\ \;+\gamma_{CIF-Mor}^{k}CIF_{d}^{k}\left(t\right)+\gamma_{ICU-Mor}^{k}ICU_{d}^{k}(t)) \end{array}$$

These differential equations are described as follows:

- Equation (1) describes arrival of suspect cases from community and cases referred from FSA to ED where they are being triaged and either will be tested, sent home or admitted to ARI/ISO/ICU based on the patient's conditions.
- The FSA flow is modeled in Equation (2) where arrivals from community and ED referrals with mild conditions will be either tested, sent home or admitted to the ARI wards. No direct admission from FSA to ISO or ICU is considered since it is assumed that FSA arrivals with relatively severe condition will be referred to ED.
- SASH flow is presented in Equation (3). The SASH program could free up healthcare resources for patients with more severe conditions.
- Equation (4) describes the ARI flow where admitted suspect cases from ED and FSA will wait for test results. Those confirmed to be COVID-19 positive will be transferred to ISO. Confirmed negative individuals will be removed directly from the ARI node. There are no confirmed COVID-19 positive and suspect cases with severe symptoms at ARI and patients with severe conditions are directly admitted to ISO/ICU from the ED.
- Equation (5a) shows the ISO flow of community cases where confirmed cases from ARI and ED admissions (for patients with relatively severe symptoms being admitted to the hospital from the CIF). Dedicated ambulances transfers were also available for positive confirmed cases from home to hospital where they are directly admitted to ISO. Stable patients from ISO can be discharged to CIF. Patients at ISO have three possible outcomes: fully recovered and discharged to home, stable but transferred to CIF or deteriorated and admitted to ICU.
- Aggressive step-down care of stable patients from hospital to external isolation facilities (e.g., CIF) (captured in this equation) was a key strategy to save hospital bed resources for severe patients and reduce mortality.
- Equation (5b) describes ISO flow of dormitory cases from various age groups where there is a direct inpatient admission of those tested positive at dormitories and require hospital care with the rate of $\gamma_{ISO}^{k}$. Similar to community cases, deteriorating dormitory patients from CIF as well as recovering patients from ICU will be transferred to ISO and stable patients from ISO will be discharged to CIF and fully recovered non-infectious cases will be discharged from ISO to home.
- Equation (6a and 6b) describes the CIF flows. The CIF compartment receives stable community and dormitory patients transferred from the hospital ISO. It also captures the referrals for deteriorating cases to either hospital ISO or ICU based on the patient's conditions. Fully recovered patients from the CIF will be discharged to home.
- Equation (7a and 7b) describe arrival of community and dormitory patients with critical conditions from ED, ISO and CIF where admitted cases have two possible outcomes of recovered and transferred to ISO or death. Direct discharge to home from ICU is not allowed in the model.
- Equation (8) shows the accumulation of recovered cases from ISO and CIF.
- Equation (9) describes the mortality outcomes from ICU. We also consider the possibility of sudden death at ISO and CIF before ICU admission in the structure of the simulation model. Notably, there were no COVID-19 deaths happened outside ICU in Singapore as of 7th Jan 2021.

Key outcome measures considered in the study were: (1) number of beds required for in-hospital and ex-hospital demands (demands that can be addressed by CIF beds outside of hospitals); (2) unmet needs considering in-hospital and ex-hospital capacities, and; (3) overall in-hospital and ex-hospital mortality rates. The types of beds that we considered were: (1) beds in isolation facilities (CIF and ISO beds) for confirmed cases; (2) beds in quarantine facilities for suspect cases, and; (3) beds in the ICUs. The model was calibrated against historical data. The time period for calibration was from 1st April till 30th April 2020. The model was then used to project demand for the period from 1st May 2020 till 31st May 2020.

### Model Variants

To adapt to the dynamic situation over time, several variants of the bed resource planning models were developed. Three of these variants are described as follows:

- *Variant 1*: This variant captured all the hospital flows of COVID patients across the multiple inpatient ward classes and external decanting facilities. However, this model did not take into account the different flows of incoming patients from dormitories and community. This variant considered the presence of both in-hospital care facilities across ARI, ISO, and ICU, as well as external care facilities with the same capabilities. External surge ARI and ISO facilities were specially setup in dedicated facilities to care for COVID cases and suspects.
- *Variant 2*: Variant 1 was extended to differentiate different incoming streams from the foreign worker dormitories and community. The model explicitly accounts for the fact that patients with mild symptoms and less risk, no existing comorbidities and younger than 40 years old would be decanted to external ISO facilities. On the other hand, patients who do not satisfy these conditions will be cared for in the ISO wards in the hospital. Consequently, the transition rates to more severe cases were assumed to be less for cases transferred to external ISO facilities (see Table 1). To better reflect the situation on the ground, we allowed for the transition of care from external ISO facilities to inpatient ISO and ICU wards. This essentially accounted for the mild cases that were earlier decanted to return to the hospital for higher levels of care. Consequently, there could be re-circulatory flows between the hospital and external isolation facilities.
- *Variant 3*: Variant 2 was extended to consider different age segments presented by the incoming dormitory flows. The consideration of age segments allows for the age-stratified risks of mild and asymptomatic cases turning symptomatic and severe, thus providing a high-resolution representation of the actual risks of over-stretching the ICU capacities in the hospital.

**Table 1:** Key parameters for the model.

**Parameter Name**	**Baseline value (Median)**	**Scenario Min**	**Scenario max**
Percentage of National Demand Coming to the SH	6%	4%	8%
**Length of Stay (LOS) in days**			
ICU	10	7	18
ISO stable^a^	4	3	7
ISO non-stable^b^	12	10	21
EISO^c^	20	17	24
ARI	2	1	2
**Case Fatality Rate (CFR)**			
ICU/EICU	8%	5%	10%
Fraction of Arrivals to SH ED	55.6%	52.3%	58.8%
Fraction of Arrivals to SH FSA	1-(Fraction of Arrivals to SH ED)		
**ED Fraction**			
ED to Hospitalization	32.5%	23.4%	45.3%
ED to SASH Fraction[estimated]	1-(ED to Hospitalization%)		
**FSA Fraction**			
FSA to Hospitalization	10.9%	4.4%	16.9%
FSA to SASH Fraction	1-(FSA to Hospitalization%)		
**Recovered Fraction**			
ICU/EICU Recovered Fraction	92%	95%	90%
ISO Stable Fraction	9.9%	5.1%	12.7%
Positive Test ARI Fraction	56.0%	39.6%	68.9%
Positive Test SASH Fraction	4.2%	1.6%	6.2%
**SH and National Capacity Estimates** ^d^			
ICU Capacity (BAU)	37	–	
ICU Capacity	70	41	189
ISO Capacity	79	73	439
ARI Capacity	269	72	440
EICU Capacity	352	310^e^	1,200
EISO Capacity (37)	40,000	10,000^f^	60,000

*SH, Study Hospital; ISO to ICU fraction, Percentage of ISO admitted cases referred to ICU; ICU-Mortality, Demised patients in ICUs; ISO-Mortality, Demised patients in Isolation Rooms; Unmet ICU, ICU demands that exceed the planned capacity (considering the surge capacity); Unmet ISO, ICU demands that exceed the planned capacity (considering the surge capacity); SH-ED Demand Coefficient, Fraction of national demand coming to SH-ED; ED Fraction, Fraction of COVID-19 suspect arrivals in ED admitted to respective locations; SH-FSA Demand Coefficient, Fraction of national demand (total daily COVID-19 suspect cases coming to healthcare system) coming to SH-FSA; FSA Fraction, Fraction of COVID-19 suspect arrivals in FSA admitted/referred to respective locations; ICU Recovered Fraction, Percentage of ICU admitted cases recovering and referred to isolation room; ISO Recovered Fraction, Percentage of ISO admitted cases recovered and discharged; ISO Stable Fraction, Percentage of stable patients at ISO that can be transferred to external ISO; Positive Test ARI Fraction, Percentage of ARI admitted cases confirmed with COVID-19 test; Positive Test SASH Fraction, Percentage of swab and discharged cases confirmed with COVID-19 test*.

a *ISO stable patients will be transferred to external ISO facilities to conserve hospital capacity for patients who require higher levels of care*.

b *ISO (non-stable) cases refer to cases that have other co-morbidities and may need to stay for a longer period in the in-hospital ISO facilities*.

c *Based on viral shedding duration reported in Zhou et al. (38)*.

d *These are ballpark estimates from internal and public information as of End March 2020. Exact numbers cannot be provided due to the confidentiality of information*

e *Estimate based on 10% downtime for EICU capacity*.

f *Assumption of only 25% of external ISO capacity can be ramped up in time due to unforeseen circumstances*.

Data was not yet readily available at the time of model building in Variant 1 and Variant 2 to confidently estimate parameters such as the LOS statistics, ICU admission rates and the CFR. Data from peer-reviewed publications complemented the local data (see Table 1). In Variant 1, 5% of patients was assumed to be critically ill, 15% to be moderately ill and the remaining 80% to be mild (38, 39). Furthermore, prevailing research also established that the majority of cases would be asymptomatic (40). These assumptions were incorporated in the model for Variant 2.

**Figure 3:**
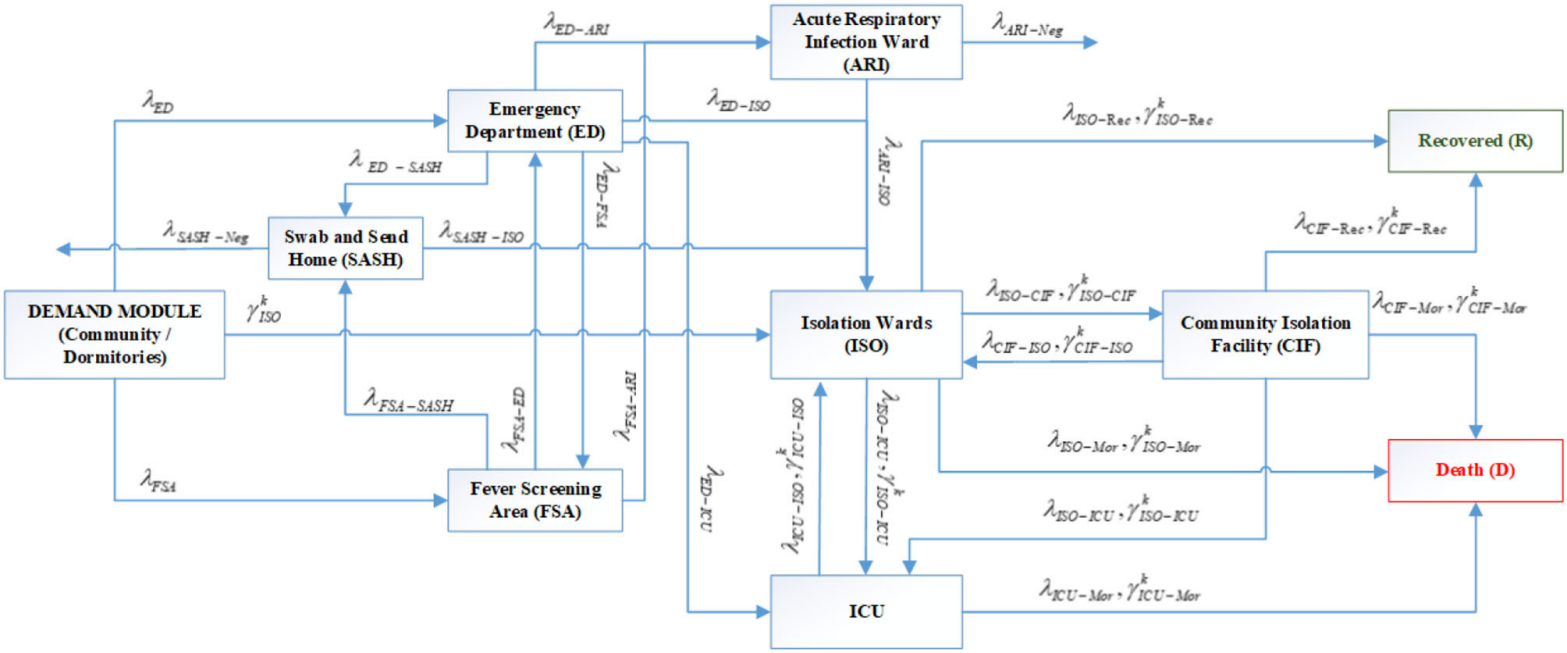
System flow schematic of Variants 2 and 3 with specialized inflows from community and dormitories in the demand module (Notations: *G*: Set of age groups, *k* ∈ *G*; λ_*i*_: Arrival rate of suspect cases from community to server *i* where *i* = {*ED*. *FSA*}; $\gamma_{ISO}^{k}$: Arrival rate of cases of age group *k* from dormitories to ISO where *k* ∈ *G*; λ_*p* − *q*_, $\gamma_{p-q}^{k}$: Transfer rate of community cases and dormitory cases of age group *k*, respectively, from server *p* to server *q* where *k* ∈ *G*, *p* = {*ED*. *FSA*.*SDC*. *ARI*. *ISO*.*CIF*. *ICU*}, *q* = {*ED*. *FSA*.*SDC*.*ARI*. *ISO*.*CIF*.*ICU*.*Rec*. *Mor*.*Neg*} and *Mor*, Mortality; *Rec*, Recovery; *Neg*, Negative confirmation for swab test).

The dual input streams from migrant dormitories and the community resulted in a bi-agent model that was deployed for Variant 2. The model structure for Variant 2 is presented in Figure 3. This variant demonstrates the differentiated inflows for patients from the community and captures the structural flows between inpatient facilities as well as external decanting facilities.

Given the unique situation that Singapore is facing with a surge, primarily due to the migrant worker population in the foreign workers' dormitories, the risk factors had to be adjusted by age groups. The age-adjusted risks of ICU admissions for the dormitory population and community were then separately estimated based on the age profiles. Variant 3 considered the following sub-groups: (1) <45 years old; (2) 45–49 years old; (3) 50–54 years old; (4) 55–59 years old, and; (5) 60 years old and above.

### Model Calibration and Sensitivity Analysis

Model calibration was made against historical data from 1 April till 30 April 2020. The models were also validated with domain experts who were involved in the planning and operations command centers in both the study hospital and the national health authorities. The models were used to project the demand for the period from 1 May 2020 till 31 May 2020. Sensitivity analysis was conducted for: (1) ICU demand coverage of 4–8% of the national population; (2) ICU median LOS from 8 to 14 days, and; (3) age-adjusted ICU conversion rate of 1–3% for the dormitory cases. For the ISO demand, sensitivity analysis was conducted for: (1) ISO demand coverage of 4–8%; (2) median in-hospital ISO LOS of 10–21 days, and; (3) median LOS of in-hospital ISO between 10–21 days and EISO of 14–24 days. Sensitivity analysis was also conducted on key parameters that would have a significant impact on capacity projections—the average length of stay (ALOS) for COVID patients in the ICU and ISO wards and the proportion of the daily national COVID demands that come to the study hospital.

### Experimental Scenarios

Projections of daily cases were made for Best-Case, Base-Case and Worst-Case scenarios. The various scenarios were estimated from a compartmental model that included the migrant workers' dormitories with further assumptions of a finite population in the migrant dormitories of ~300,000 migrant workers (36, 41). The underlying infection dynamics governing the rise in cases were assumed to be similar except for the time to reach the apex of the infection curve. For the Best-Case scenario, the apex was assumed to have been reached in the third week of April 2020. For the Base Case, the apex was projected to be reached in the start of May 2020 and for the Worst Case. For the Worst Case, the apex was estimated to be toward the end of May 2020. The projections started when the surge in infections occurred at the end of April 2020 (See Figure 3 for both the projected number of daily cases and the cumulative number of cases across the 3 scenarios) and were constantly updated with available data as the pandemic evolved.

## Results

### Model Calibration and Sensitivity Analysis

**Figure 4:**
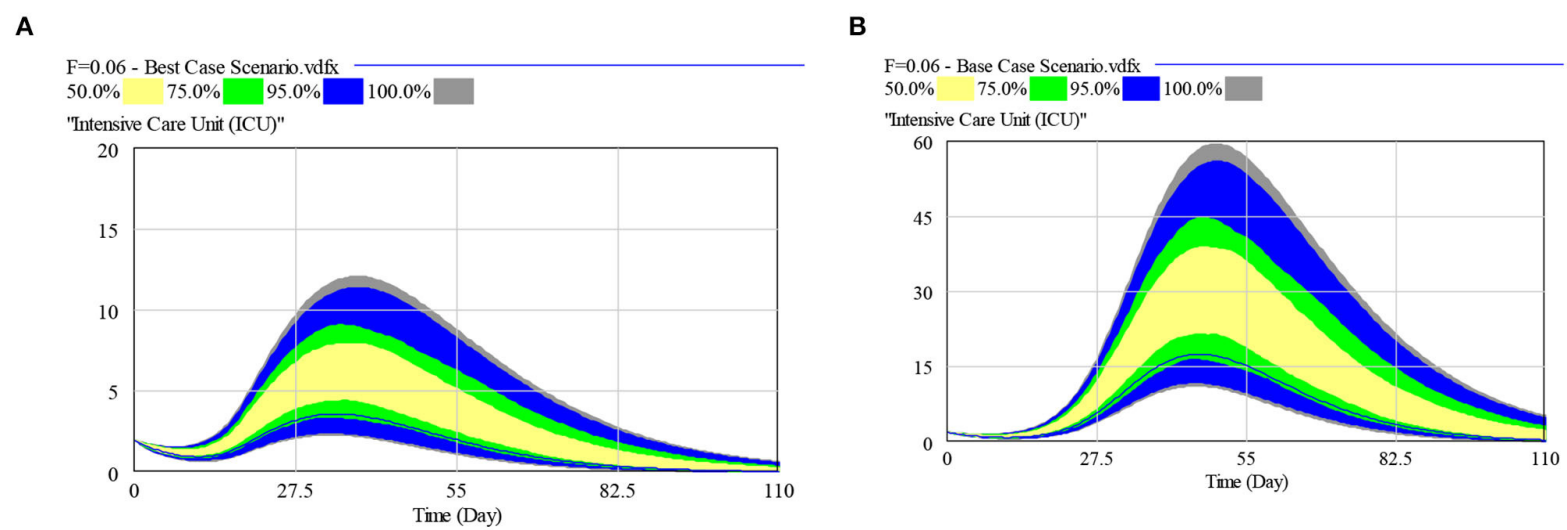
Multivariate sensitivity analysis for: **(A)** Best Case ICU beds requirements, and **(B)** Base Case ICU beds requirements [Assumptions: SH with a national demand coverage of 4–8% and median ICU LOS of 7–18 days].

The model calibration curves closely matched the historical trends and tracked the rapidly evolving dynamics, from stabilizing community infection to the surge predominantly arising within the dormitories. More than 90% of the cases were projected and observed to be from the migrant dormitories. The projections based on the bi-agent model (including the time to peak for ICU and ISO bed needs for the SH and national demands) with percentiles of the sensitivity analyses for the baseline, best, base and worst cases are listed in Table 2. Sensitivity analysis results for the bed demands, assuming 4–8% and median ICU LOS of 7–18 days, are shown in Figure 4 for the best and base case parameters for ICU beds requirements of the SH.

**Table 2:** Projections based on the bi-agent model (Variant 3) across Best, Base and Worst Cases and their planning capacities.

**Study Hospital/National Capacity**	**Type of Beds**	**Required Capacity at Peak**				**Time to Peak [Best, Base, Worst Case]**
		**[Best, Base, Worst Case]**	**Best Case Percentiles [2.5%, 97.5%]**	**Base Case Percentiles [2.5%, 97.5%]**	**Worst Case Percentiles [2.5%, 97.5%]**	
SH	ICU	[4, 18, 33]	[2, 12]	[13, 57]	[20, 105]	[35, 46, 52]
	Non-ICU (ISO)	[240, 405, 685]	[190, 406]	[295, 700]	[550, 1,190]	[36, 45, 52]
National	ICU	[60, 290, 540]	[40, 135]	[197, 650]	[375, 1,210]	[35, 46, 52]
	Non-ICU (ISO)	[18,680, 30,885, 49,925]	[18,000, 20,000]	[29,000, 33,000]	[48,000, 53,000]	[30, 38, 43]
**Planning Capacities**						
SH	ICU Planning Capacity (Baseline, Min, Max): [70, 41, 189]					
	Non-ICU (ISO) Planning Capacity (Baseline, Min, Max): [79, 73, 439]					
National	ICU Planning Capacity (Baseline, Min, Max): [352, 310, 1,200]					
	Non-ICU (ISO) Planning Capacity (Baseline, Min, Max): [40,000, 10,000, 60,000]					

### Age-Dependent Risks

**Figure 5:**
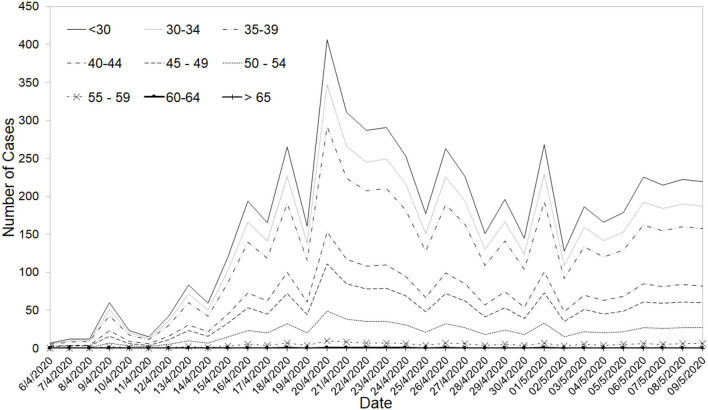
Distribution of the number of cases across age groups for the migrant dormitories' cases.

During the study time period, age-dependent risks on the severity of COVID-19 patients have already been reported in several existing studies (16, 39, 42). Estimates of these age-dependent risks for the Singapore population were derived from an unpublished study by the National Centre for Infectious Diseases in Singapore based on data of 1,481 patients were utilized to develop Variant 3. The risk of patients requiring ICU ranged from 0% for the population below 30 years old to 19.45% for patients who are 65 years old and above. The age-adjusted ICU conversion rate was then determined to be 1.55% for the dormitory and 4.95% for the community stream. The age profile of dormitory cases is also different from the age profile of community cases (see Figure 5). The bi-agent model was re-calibrated with the new age-adjusted risk assumptions.

### Summary of Results

**Figure 6:**
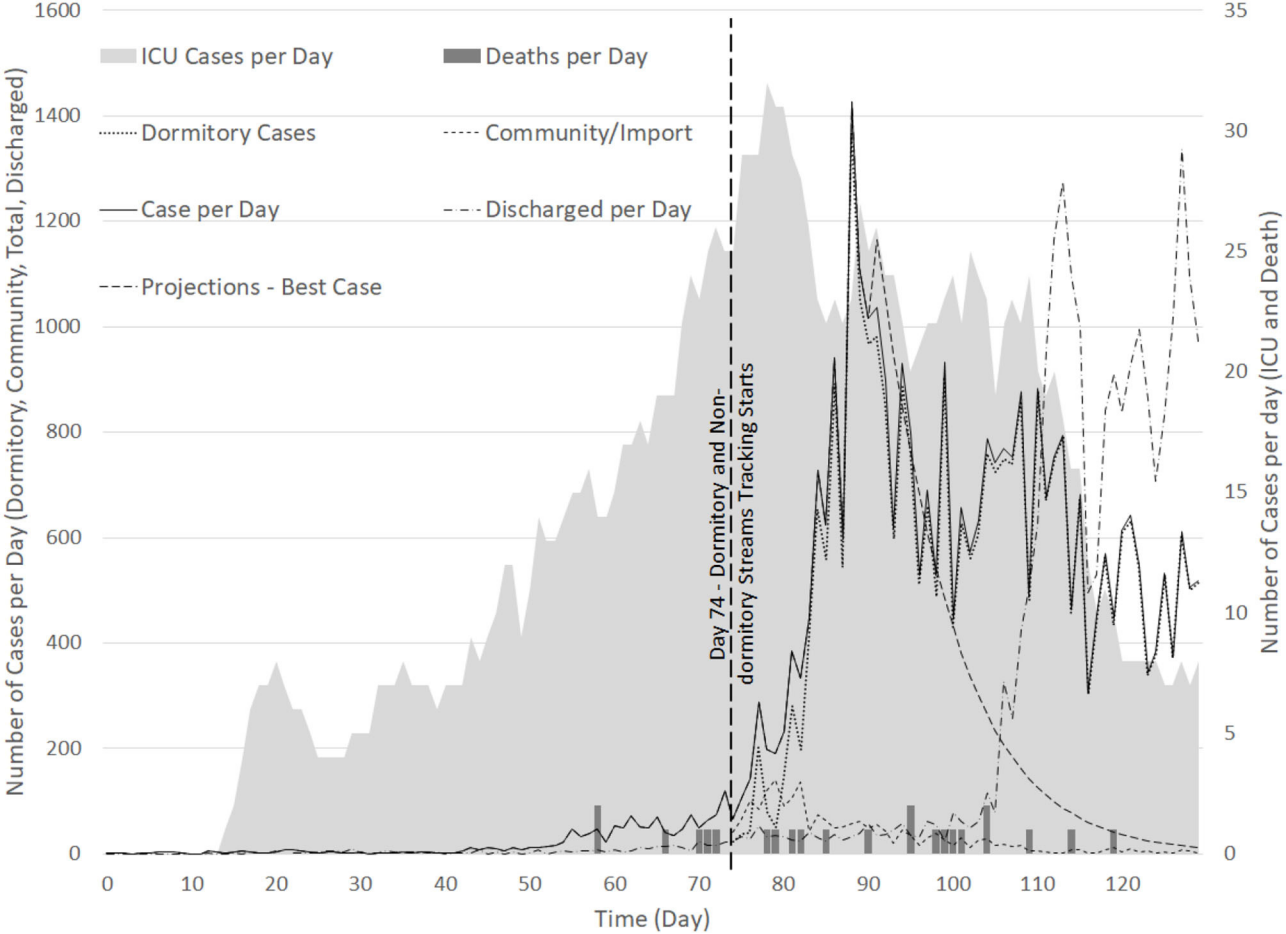
Time series of cases detected per day for (1) Dormitory cases (from Day 74); (2) Community cases (from Day 74); (3) Total cases; (4) ICU cases, and; (5) Deaths.

The SH has around 1,800 beds, with 18 ICU beds, in 2019. As the surge of cases from the dormitories evolved to be the dominant stream, tracking for dormitory cases was started only from 6 April (or Day 74) (see Figure 6). The number of cases from the dormitories were observed to increase steadily from 6 April 2020 and peaked at 1,426 cases on 20th April 2020, declining thereafter (32). As of 21 April 2020, there were 3,566 COVID-19 patients in nationwide hospital isolation wards and 27 in intensive care, translating to about 31.7% of inpatient hospital beds used for COVID-19 patients. During the period from 1st till 20th April 2020, 1,589 SASH were carried out from the total of 2,189 suspect assessments. Out of the 600 cases admitted, 404 (37.10%) and 196 (17.82%) admission decisions were made at ED and FSA, respectively.

The model was built in the initial stages of pandemic preparedness period when the DORSCON level was raised to “Orange” (43). After the surge in infections, the team quickly pivoted to consider a bi-agent model to take into consideration the stream of infections from the dormitory clusters, separated from the community cluster. With the updated bi-agent model, we were able to differentiate the unique stream from the dormitories which grew from 6th April till 6th May 2020 and stabilized at an average of 98.8% (IQR: 0.11%) (from 7th till 31st May 2020) of the infected population. The later variant of the model accounted for the rapidly changing bed resource management policies both for in-hospital and external isolation facilities. There had also been a number of other dynamic adjustments made in terms of the detection, diagnosis and disposition policies for COVID-19. These included the expansion of PCR swab test policies for high-risk groups in the dormitories and community (33) and heightened surveillance of the population (33) apart from the agile flexing of in-hospital capacities and the swift ramp-up of external isolation facilities (34).

The modeling framework supported high-resolution bed resource planning decisions for the short term (across weeks). In anticipation of the surge in demand prior to the migrant dormitory worker outbreak, the SH had progressively increased its bed capacities. The hospital had also made surge plans for ICU capacity from a pre-COVID level of around 40 beds to ~200 beds when needed. These additional surge capacities for ICU require setup times ranging from 1 day to 15 days to ramp up. Consequently, plans have to be established at least 2 weeks in advance to prepare for any potential surge in infections. The model with the ability to support planning across weeks supported the decision-making process.

## Discussion

During the early stages of the pandemic, the lack of scientific knowledge regarding the SARS-CoV2 beta-coronavirus resulted in difficulties with accurate forward predictions regarding the pandemic. Due to inherent uncertainties, any predictive models must be able to learn from new data, and the health system must have the capability to assimilate new knowledge, plan and respond effectively in dealing with emerging infectious diseases. Health system resilience, which is the capacity of health actors, institutions, and populations to prepare for and effectively respond to crises whilst maintaining core functions (44), is crucial in the sustainable delivery of care by the health system. This study is a first step toward a framework to achieve the goals of a resilient healthcare system (45, 46) with the ability to support the design of discharge policies and bed surge capacities under significant uncertainties in a novel pandemic.

### Discharge Policies

As of 12 May 2020, ~3,900 PCR tests per hundred thousand people in Singapore were conducted (32). External evidence has shown that viable viral replication drops rapidly after 7–10 days from the onset of symptom (47). Based on preliminary analysis of 766 COVID-19 cases, it was estimated then that the duration of viral shedding through PCR tests via nasopharyngeal swabs could be longer than 33 days for ~5% of all confirmed cases (48). Consequently, it was determined that de-isolation and discharge policies should not depend solely on the viral ribonucleic acid (RNA) detection via PCR tests (48). More aggressive discharge of patients based on the evidence presented at the time of the study and other clinical parameters, rather than PCR results, were subsequently considered. This led to a higher discharge rate of patients from Day 110 as shown in Figure 6, and better focus of the healthcare bed resources to care for patients with early diagnosis, quarantine and treatment (48).

### Bed Resources

By working in tandem with external isolation facilities run by the government and private hospitals, the modeling results showed that the swift decanting of Covid-19 patients with mild symptoms to external CIFs had further prevented the over-congestion of hospital capacities. Model results showed that the SH and national ICU capacities were well-prepared to deal with the worst-case demand projections at the 97.5th percentile level against the uncertain parameters. For the non-ICU beds, the planning capacity also appeared to be sufficient to deal for the best-case scenario in the hospital's bed demands, and the base case scenario for the national ISO bed demands (which included the external isolation facilities). By maintaining the sufficiency of healthcare capacity with a respectable safety margin, Singapore was able to keep the CFR to be amongst the lowest in the world (49).

### Uncertainties in Planning

The proposed modeling framework was able to deal with: (1) demand side uncertainties related to the uncertain pandemic scenarios, evolving detection and disposition (50); (2) supply side uncertainties related to dynamic bed capacity management including the bed capacities of in-hospital and external isolation facilities; (3) parametric uncertainties related to the lack of precise and accurate estimation of key parameters required for modeling, and; (4) a collaborative framework that facilitates the development of the model and assimilation of insights by key stakeholders. The proposed modeling framework is agile, adaptable and supported by a strong ecosystem to facilitate the assimilation of new data and knowledge for evidence-based decision support. The presence of robust communication channels throughout the health system further ensured timely and accurate dissemination of modeling insights. With the ability to continuously learn and calibrate responses with new data and knowledge, the framework facilitated rapid reorganization and adaptation to achieve a resilient health system (44, 51).

### Supporting Infrastructure and Collaborative Efforts

The database architecture within the AMC has already been earlier established and was able to effectively support the COVID-19 pandemic response modeling exercise. Furthermore, the core data science expertise embedded in the health system had the necessary domain knowledge to pool together the set of real-world data from the various source systems and the enterprise data warehouse (EDW). These data sources were critical for the model to be updated with the latest projections. Clinical risk factors that have been considered in the patient disposition at the time of the study included age, chronic comorbidities (diabetes mellitus, heart, lung and kidney diseases), supplementary oxygen needs, clinical features (e.g., dyspnoea, respiratory rates and SpO_2_ levels), chest X-rays and laboratory results. The empirical data showed limited risks of patients turning severe to require higher levels of care (e.g., in-hospital beds and ICUs). To achieve more robust insights, sensitivity analyses were performed to evaluate the bed capacity needs (ICU and external vs. in-hospital conversion rates and the LOS in these different facilities) across the best, base and worst- case scenarios. The projections and sensitivity analysis provided useful inputs for the policy makers and resource planners.

Rapid health systems modeling during pandemics requires a strong collaborative effort amongst a wide variety of stakeholders (52, 53). The modeling team in this study comprised of senior clinicians from the emergency medicine, critical care medicine, infectious disease, epidemiological and health services research domains. The modeling expertise came from disciplines ranging from industrial engineering, computer scientists and biostatisticians within the AMC. The formal organization structures established since the beginning of the outbreak (e.g., health system's disease outbreak task force, the critical care and ICU planning team, the operating theater and bed management unit) facilitated the rapid dissemination of study results. All these factors were critical considerations for the realization of a resilient healthcare system during pandemics (45).

### Limitations

The models were built to specifically address the bed management policies in a Singapore public hospital and may not be generalizable to other health systems. Nonetheless, the modeling framework can be customized to consider different types of bed resources, patient demands, and process flows. Moving forward, the dynamic hypotheses captured by the current models have to continually evolve over time for different use cases. Even as we see declining cases in Singapore, sporadic numbers of community and imported cases have been detected. As countries start to gradually exit from lockdowns in phases and transnational travel is revived (54), the risk of new waves of infections is a ongoing concern (55).

## Conclusion

This study showcases a modeling framework that was successfully deployed in Singapore for bed resource planning during the early phases of the COVID-19 pandemic. The rapidly evolving pandemic with the detection of new variants and infection sources necessitated the development of an agile modeling framework. The framework provides a platform for decision-makers to quickly evaluate complex systems trade-offs to support bed resource planning during pandemics in a holistic manner. The study has also shown that the tightly integrated nature of the Singapore healthcare system is important to enable close coordination and timely information sharing across diverse groups of stakeholders and decision makers.

## Data Availability Statement

Updated data and model results is available on http://hi-board.ap-southeast-1.elasticbeanstalk.com/. Username and password to access this site can be made available upon request to the Corresponding Author. Additionally, the Singapore COVID-19 national datasets used for this study can be found in the Singapore Ministry of Health COVID-19 Updates on https://www.moh.gov.sg/covid-19.

## Ethics Statement

The studies involving human participants were reviewed and approved by Centralized Institutional Review Board of the Singapore Health Services (opinion date 26 May 2020, number 2020/2470). Written informed consent from the participants' legal guardian/next of kin was not required to participate in this study in accordance with the national legislation and the institutional requirements.

## Author Contributions

SL, AP, HA, JA, DM, and MO were responsible for the conception and study design. SL, AP, and FN did the literature review, modeling, data analysis, and drafting the manuscript. SL, AP, and MO made significant revisions. SL, DM, and MO supervised the analysis, modeling, and the interpretation of data. FS, JA, JL, DM, and MO supplied valuable improvement suggestions to the analysis and the manuscript. MO is the principal investigator of the overall NMRC grant which provided the funding for this research. All authors contributed to the article and approved the submitted version.

## Funding

This project was funded by the COVID-19 Research Fund from the National Medical Research Council (NMRC) of the Ministry of Health (MOH), Singapore grant (COVID19RF2-0028). The study results were used to inform the MOH on the bed resource management decisions in during the COVID-19 pandemic.

## Conflict of Interest

The authors declare that the research was conducted in the absence of any commercial or financial relationships that could be construed as a potential conflict of interest.

## Publisher's Note

All claims expressed in this article are solely those of the authors and do not necessarily represent those of their affiliated organizations, or those of the publisher, the editors and the reviewers. Any product that may be evaluated in this article, or claim that may be made by its manufacturer, is not guaranteed or endorsed by the publisher.

## Abbreviations

ALOS: Average Length-of-stay
AMC: Academic Medical Centre
ARI: Acute Respiratory Infection beds
BAU: Business-as-usual
CFR: Case Fatality Rate
CIF: Community Isolation Facility
DES: Discrete Events Simulation
DORSCON: Disease Outbreak Response System Condition
ED: Emergency Department
EDW: Enterprise Data Warehouse
EICU: External ICU beds
EISO: External ISO beds
FSA: Fever Screening Area
ICU: Intensive Care Unit
IQR: Interquartile Range
ISO: Isolation beds
LOS: Length-of-stay
MOH: Ministry of Health, Singapore
NCID: National Centre for Infectious Diseases
PCR: Polymerase Chain Reaction
SASH: Swab and Send Home
SD: System Dynamics
SGH: Singapore General Hospital
SH: Study Hospital
SingHealth: Singapore Health Services.
